# Multiple tasks and neuroimaging modalities increase the likelihood of detecting covert awareness in patients with disorders of consciousness

**DOI:** 10.3389/fnhum.2014.00950

**Published:** 2014-11-26

**Authors:** Raechelle M. Gibson, Davinia Fernández-Espejo, Laura E. Gonzalez-Lara, Benjamin Y. Kwan, Donald H. Lee, Adrian M. Owen, Damian Cruse

**Affiliations:** ^1^The Brain and Mind Institute, University of Western OntarioLondon, ON, Canada; ^2^Department of Psychology, University of Western OntarioLondon, ON, Canada; ^3^Department of Medical Imaging, University of Western OntarioLondon, ON, Canada; ^4^Department of Radiology, London Health Sciences CentreLondon, ON, Canada

**Keywords:** disorders of consciousness, vegetative state, neuroimaging, electroencephalography, mental imagery, motor imagery

## Abstract

Minimal or inconsistent behavioral responses to command make it challenging to accurately diagnose the level of awareness of a patient with a Disorder of consciousness (DOC). By identifying markers of mental imagery being covertly performed to command, functional neuroimaging (fMRI), electroencephalography (EEG) has shown that some of these patients are aware despite their lack of behavioral responsiveness. We report the findings of behavioral, fMRI, and EEG approaches to detecting command-following in a group of patients with DOC. From an initial sample of 14 patients, complete data across all tasks was obtained in six cases. Behavioral evaluations were performed with the Coma Recovery Scale—Revised. Both fMRI and EEG evaluations involved the completion of previously validated mental imagery tasks—i.e., motor imagery (EEG and fMRI) and spatial navigation imagery (fMRI). One patient exhibited statistically significant evidence of motor imagery in both the fMRI and EEG tasks, despite being unable to follow commands behaviorally. Two behaviorally non-responsive patients produced appropriate activation during the spatial navigation fMRI task. However, neither of these patients successfully completed the motor imagery tasks, likely due to specific motor area damage in at least one of these cases. A further patient demonstrated command following only in the EEG motor imagery task, and two patients did not demonstrate command following in any of the behavioral, EEG, or fMRI assessments. Due to the heterogeneity of etiology and pathology in this group, DOC patients vary in terms of their suitability for some forms of neuroimaging, the preservation of specific neural structures, and the cognitive resources that may be available to them. Assessments of a range of cognitive abilities supported by spatially-distinct brain regions and indexed by multiple neural signatures are therefore required in order to accurately characterize a patient's level of residual cognition and awareness.

## Introduction

Disorders of consciousness (DOC), such as the Vegetative State (VS) and Minimally Conscious State (MCS), are diagnosed on the basis of behavioral responses to external stimulation (Jennett, 2002; Owen, 2008). Patients in the VS exhibit periods of eye opening and eye closing that resemble the sleep-wake cycles of fully awake and aware individuals (Multi-Society Task Force on PVS, 1994a,b; Cruse et al., 2013). Critically, however, patients in the VS do not engage in any purposeful outward responses to verbal commands (Jennett, 2002; Owen, 2008). In contrast, patients diagnosed as in a MCS show some reproducible markers of awareness and responsiveness to external stimulation (Giacino et al., 2002; Owen, 2008). It has been proposed that patients in a MCS be sub-categorized into two groups (MCS *Plus* and MCS *Minus*) based on the complexity of their observed behavioral responses (Bruno et al., 2011, 2012). Patients sub-categorized as in a MCS *Plus* demonstrate at least one of command-following, intelligible verbalization, or verbal or gestural yes/no responses, while patients sub-categorized as in a MCS *Minus* show only minimal levels of behavioral interactions via non-reflexive movements (Bruno et al., 2011, 2012). In addition to this gradient of responsiveness among patients with DOC, the behavioral evaluation of patients with DOC is confounded by the fact that the pattern of brain injury may include a compromised peripheral motor system. It is therefore plausible that, as a result of impaired motor abilities, a patient who retains awareness and cognitive function could be inappropriately identified as VS (Owen, 2008, 2013).

Functional neuroimaging provides one means to detect cognitive functions without relying on external behavior. In 2006, Owen and colleagues used functional Magnetic Resonance Imaging (fMRI) to determine whether a patient diagnosed as in the VS could follow spoken commands by modulating her brain activity rather than producing overt movements (Owen et al., 2006). The patient was asked to perform two types of mental imagery during the fMRI scan: (1) to imagine playing tennis, a motor-imagery task which elicits activation in the supplementary motor area in non-brain-injured volunteers, and (2) to imagine moving through the rooms in her house, a task which elicits activation in a network of regions including the parahippocampal gyrus, the posterior parietal cortex, and the lateral premotor cortex. Remarkably, this patient produced brain responses that were nearly indistinguishable from non-brain-injured volunteers for both imagery tasks. This finding provided strong evidence that the patient was capable of following commands, and was therefore aware despite her behavioral profile (Owen et al., 2006). Mental imagery paradigms and related tasks have since demonstrated that a significant minority of patients who are diagnosed as in the VS can follow (Owen et al., 2006; Monti et al., 2010; Bardin et al., 2011; Cruse et al., 2011; Goldfine et al., 2011; Naci and Owen, 2013), or attempt to follow (Bekinschtein et al., 2011; Cruse et al., 2012) commands by modulating their fMRI-detected brain activity.

Another neuroimaging technique that has been used to assess residual cognition in patients with DOC is electroencephalography (EEG). EEG is readily available in clinical environments due to its regular use to assess, for example, epileptiform activity, and prognosis in coma (Wijdicks et al., 2006). Crucially, EEG assessments can be performed at the patient's bedside. As with fMRI, EEG researchers have used mental imagery to assess residual cognition in patients with DOC. Motor imagery—i.e., imagining movement—is reflected in the EEG by decreases (also known as an event-related desynchronizations, or ERDs) and/or increases (event-related synchronizations, or ERSs) in spectral power in the mu (7–13 Hz) and beta (13–30 Hz) frequency bands (Pfurtscheller and Neuper, 1997; Pfurtscheller and Lopes da Silva, 1999). While there is variation across individuals (Cruse et al., 2012; Gibson et al., 2013), motor imagery ERDs often occur over the contralateral sensorimotor cortex (e.g., left sensorimotor cortex for imagined movements of the right hand, etc.; Pfurtscheller and Neuper, 1997; Pfurtscheller and Lopes da Silva, 1999). As with the fMRI assessments, task-appropriate motor imagery responses have been observed in the EEG of a small number of patients with DOC, indicating that these patients were capable of following commands even though they were unable to do so with their external behavior (Bekinschtein et al., 2011; Cruse et al., 2011, 2012; Goldfine et al., 2011).

A key challenge when detecting covert awareness is that a DOC can result from a wide range of etiologies. Patients with DOC may have traumatic or non-traumatic brain injuries, and comorbidity with other disorders and pathologies is common. Accordingly, there is likely to be high variability between individuals in the specific cognitive abilities that may be preserved. Furthermore, variable behavioral abilities within patients are common across relatively short time-frames (Giacino et al., 2002; Cruse et al., 2013). Some patients will also be ineligible for certain types of neuroimaging. For example, metallic implants may be incompatible with MRI, and craniotomies can result in highly abnormal EEG recordings (Lee et al., 2010). For these reasons, it is critical to utilize multiple assessment techniques (e.g., behavior, fMRI, EEG, etc.) with a range of cognitive and sensory tasks in order to obtain an accurate representation of a patient's abilities.

In the current manuscript, we report for the first time the outcomes of behavioral evaluations and well-known fMRI and EEG protocols in a small group of DOC patients. Behavioral assessments were performed using the Coma Recovery Scale-Revised (CRS-R) (Kalmar and Giacino, 2005). In the fMRI assesment, patients were asked to perform motor imagery (playing tennis) and spatial navigation imagery (moving through a familiar place) using the paradigm reported in previous work with patients with DOC (Owen et al., 2006; Monti et al., 2010; Fernández-Espejo and Owen, 2013; Fernández-Espejo et al., 2014) and non-brain-injured volunteers (e.g., Boly et al., 2007). In the EEG assesment, patients were asked to perform two types of motor imagery: squeezes of the right-hand (“conventional motor imagery”) and an action with which they had experience prior to their injury (“familiar motor imagery”). Hand squeezes were included in the EEG assessment because this type of action is widely used in EEG motor imagery tasks, including tasks for patients with DOC (e.g., Cruse et al., 2011, 2012; Goldfine et al., 2011). The secondary familiar imagery task was included following the recommendations of previous work with non-brain-injured volunteers that indicated the potential for higher sensitivity relative to conventional hand squeezing (Curran and Stokes, 2003; Curran et al., 2004; Gibson et al., 2013).

## Materials and methods

### Patients

An initial convenience sample of 14 patients with severe brain injury and DOC diagnoses ranging from MCS (*Plus*) to VS were recruited for the EEG and fMRI tasks. Surrogate decision makers provided written informed consent for each of the patients in both the EEG and fMRI studies. Ethical approval was obtained from Western University's Health Sciences Research Ethics Board. Three patients were excluded from the sample because they were ineligible for the fMRI assessment; two patients were excluded because they had craniotomies that resulted in poor quality EEG data (Lee et al., 2010); and three other patients were excluded due to excessive movement artifacts. The remaining sample of six patients included three patients in the VS and three patients in a MCS. Five patients (Patients 2–6) completed the fMRI and EEG experimental procedures in the same week with 1–3 days between sessions, and one patient (Patient 1) completed the fMRI experimental procedure 7 months prior to the EEG experimental procedure. In the latter case (Patient 1), the patient's ability to follow commands using neuroimaging-based assessments has been previously documented using the same fMRI mental imagery described here (Fernández-Espejo and Owen, 2013), an fMRI-based attentional paradigm (Naci and Owen, 2013), and an EEG attempted movement paradigm (Cruse et al., 2012). Demographic and clinical data for the final sample of patients is included in Table 1.

**Table 1:** **Patients' demographic and clinical assessment data**.

**Patient No**.	**Sex**	**Age (y)**	**Interval since Ictus (y)**	**Etiology**	**Diagnosis**	**CRS-R Score^a^**
1	M	38	13 (fMRI)	*Traumatic*	Vegetative state	7
			13.6 (EEG)	Traumatic brain injury secondary to a motor vehicle collision		
2	F	20	6	*Non-Traumatic*	Vegetative state	8
				Undiagnosed progressive neuromuscular deterioration		
3	M	27	4	*Non-Traumatic*	Minimally conscious state *Plus*^b^	13
				Anoxic brain injury secondary to cardiac arrest		
4	F	46	20	*Non-Traumatic*	Minimally conscious state *Minus*	10
				Hypoxic brain injury due to drowning		
5	M	57	4	*Non-Traumatic*	Vegetative state	6
				Diffuse anoxic brain injury secondary to cardiac arrest		
6	F	35	2	*Non-Traumatic*	Vegetative state	5
				Anoxic brain injury secondary to bilateral pulmonary emboli and cardiac arrest		

*Abbreviations: fMRI, functional Magnetic Resonance Imaging; EEG, electroencephalography; CRS-R, Coma Recovery Scaled-Revised*.

a *Highest CRS-R score recorded by the research team until the time of assessment. For Patients 2-6, this period was three to nine months (see text for details). For Patient 1, this period was 24 months (21 evaluations)*.

b *Patient 3 generated reproducible movements to spoken commands on the auditory sub-scale of the CRS-R on each evaluation*.

### Imagery tasks

During the fMRI testing sessions, patients were asked to perform alternating sessions of repeated rest-imagery cycles. Each period of imagery or rest lasted for 30 s, and each patient completed five cycles for both imagery tasks. In the motor imagery task, participants were instructed to imagine swinging an arm to hit a tennis ball in a tennis match. In the spatial navigation task, they were instructed to imagine walking from room to room in their house and visualize all objects they would encounter if they were in their home. The experimental procedure has been reported in previous work (Owen et al., 2006; Monti et al., 2010; Fernández-Espejo et al., 2014).

For the EEG task, the procedure was similar to that reported in Cruse et al. (2012); Gibson et al. (2013). Specifically, every trial began with one of three instructions: “Imagine squeezing your right-hand,” “Imagine dialing 9-1-1” (or a custom action, detailed in Supplementary Table 1), and “Now, please just relax.” All instructions were 3-s in length and were followed by 2- to 5-s of silence. The silent interval was selected randomly from a uniform distribution on each trial, and the instructions were presented by earphone. The task was completed in blocks of 48 trials (16 trials per instruction) presented in a pseudorandom order; no more than three instructions of the same type were presented consecutively. Each patient completed four or five blocks during the assessment, for a total of 192 (four blocks) or 240 (five blocks) trials, with short breaks between each block.

### fMRI data acquisition and analysis

fMRI data were acquired in a 3 Tesla Siemens scanner (Magnetom Trio Tim, Siemens, Germany) with a Siemens 32-channel head-coil (Patients 2, 3, and 6) or a Siemens 12-channel head-coil (Patients 1, 4, and 5) at the Centre for Functional and Metabolic Mapping at Robarts Research Institute, Western University, Canada. Head-coils were chosen on a patient per patient basis to ensure their comfort. The MRI protocol included a single session of 165 volumes, of 36 axial slices each covering the whole brain, using echo-planar images (repetition time = 2000 ms, echo time = 30 ms, matrix size = 70 × 70, slice thickness = 3 mm, in-plane resolution = 3 × 3 mm, flip angle = 78°). High-resolution T1-weighted 3D MP-RAGE images (repetition time = 2300 ms, echo time = 2.98 ms, inversion time = 900, matrix size = 256 × 240, voxel size 1 × 1 × 1 mm, flip angle = 9°) were acquired in the same session. The task instructions and cues were presented using E-Prime® 2.0 running on Windows XP on an iMac computer and an MRI-compatible high-quality digital sound system incorporating noise-attenuated headphones (Silent Scan™, Avotec Inc.).

The fMRI data were pre-processed and analyzed using SPM8 (http://www.fil.ion.ucl.ac.uk/spm). Data were first manually AC-PC reoriented. Spatial pre-processing included: realignment to correct subjects' motion, co-registration between the structural and functional data sets, and smoothing with an 8-mm full width at half maximum Gaussian kernel. Single subject fixed-effect analyses were performed in each patient. The analysis was based on the general linear model using the canonical hemodynamic response function (Friston et al., 1995). Each scan was modeled as belonging to the mental imagery (i.e., motor imagery or spatial navigation) or the rest condition. Movement parameters calculated from the realignment step were also included as covariates of non-interest. Additionally, we discarded repetition times with levels of motion above 2 mm and 0.035 rad. High-pass filtering using a cut-off period of 128 s was implemented in order to remove slow-signal drifts from the time series. Linear contrasts were used to obtain subject-specific estimates of each of the effects of interest. Results were thresholded at a voxel level family-wise error (FWE) whole-brain *p* < 0.05. In healthy volunteers, spatial navigation imagery is typically associated with strong and reliable activity in the parahippocampal gyrus, the posterior parietal cortex, and the lateral premotor cortex, while tennis imagery elicits activity in the supplementary motor area (Boly et al., 2007; Fernández-Espejo et al., 2014). We have included supplementary figures from a previous study (Fernández-Espejo et al., 2014) depicting single-subject activation in a sample of 14 healthy young adults for spatial navigation imagery (Supplementary Figure 1) and tennis imagery (Supplementary Figure 2). Because of our strong anatomical *a priori* hypotheses, when no significant activations were found at this level we reduced the statistical threshold to an uncorrected *p* < 0.001 to exclude the possibility of failing to detect more subtle changes in the blood oxygen level-dependent signal due to this conservative approach (Friston et al., 1996; Fernández-Espejo et al., 2010).

### EEG data acquisition and analysis

EEG data were recorded using the g.Gamma active electrode system (g.tec Medical Engineering GmbH, Austria) with a four-channel montage housed in an electrode cap. The electrodes were placed at sites CP3, FC3, CP4, and FC4, and the EEG signals were acquired using a g.USBamp amplifier. Stimuli presentation and physiological data recordings were performed using a Simulink® model in MATLAB® (The Mathworks, Inc., Natick, MA). Online, the EEG data were filtered from 0.5 to 60 Hz with a 60 Hz notch filter. The recordings were referenced to the right earlobe with a forehead (Fpz) ground. The data were sampled at 600 Hz with impedances kept below 5 kΩ. Offline, the EEG data were down-sampled to 100 Hz, filtered between 0.5 and 40 Hz, and segmented into 6-s epochs time-locked to the onset of the auditory cue. Trials containing physiological artifacts were identified by visual inspection and removed. After artifact rejection, the median number of trials included in each imagery and rest condition per patient was: Movement 1 (hand squeeze) – 43 (range: 29–57); Movement 2 (custom) – 45 (range: 32–57); and Rest – 44 (range: 27–58). Finally, the EEG data were re-referenced offline to form two bipolar channels (FC3 – CP3, FC4 – CP4) that are subsequently identified as C3′ and C4′, respectively; this bipolar approach is known to detect changes in mu (7–13 Hz) and beta (13–30 Hz) power with high accuracy across many people (Cruse et al., 2012).

The EEG data were analyzed from 7 to 30 Hz using the same spectral analysis procedure reported in previous work (Cruse et al., 2012; Gibson et al., 2013). We have provided a supplementary figure (Supplementary Figure 3) depicting patterns of spectral changes from a sample of six healthy young adults using the same task and analysis procedure from Cruse et al. (2012). For each time-point at C3′ and C4′, spectral power estimates were calculated using a Hanning window (1-s) time-frequency transformation via the “ft_freqstatistics” function from the open-source MATLAB toolbox, FieldTrip (Oostenveld et al., 2011). The time-frequency data at both electrodes were then compared between motor imagery and rest using cluster-based permutation testing (*cf*. Maris and Oostenveld, 2007; Cruse et al., 2012; Gibson et al., 2013). For the cluster-based testing, the time-frequency data for each imagery condition and rest were log-transformed and then compared at each data point using paired-samples *t*-tests. All significant data points (*p* < 0.025) were then arranged into clusters based on their temporal and spectral proximity to each other, and the *t*-values were summed for each cluster. A Monte Carlo randomization test that controlled for FWE was used to determine the significance value for each cluster. In the randomization test, the condition labels were randomly permuted, and the clustering procedure was repeated 1000 times. The maximum summed *t*-value clusters from each repetition were used to form a distribution, and this distribution was then used to test the null hypothesis that the original summed *t*-value occurred by chance.

## Results

**Figure 1:**
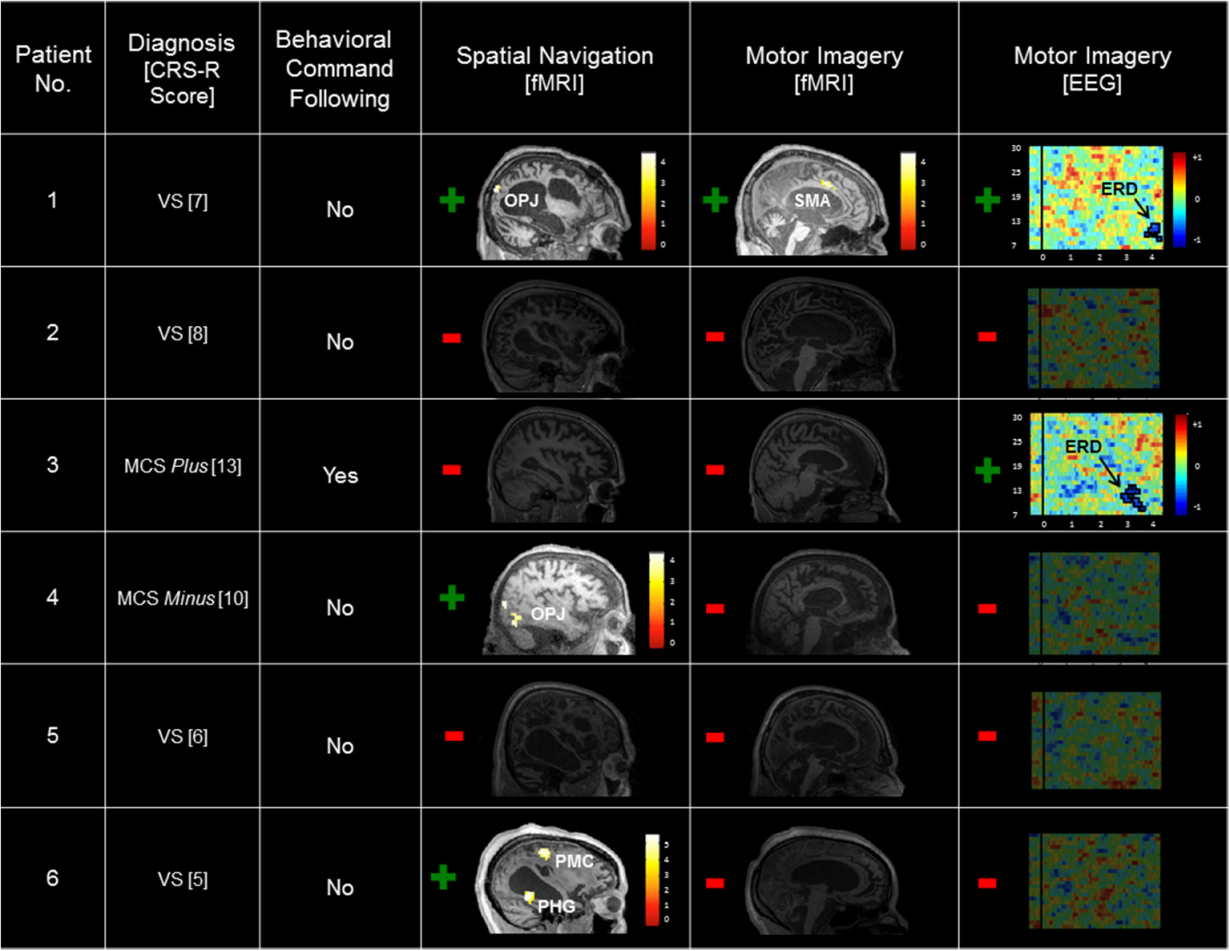
**Summary of patient results across the behavioral, fMRI, and EEG assessments**. Significant task-related fMRI activation is labeled by region. Scales depicting the *t*-value statistical maps are inset, and results are thresholded at an uncorrected *p* < 0.001 and rendered on each patient's T1 MRI image for display. Spectrograms of the log ratio differences in EEG power between conventional motor imagery and rest are shown for the left (contralateral) hemisphere. The vertical axis depicts the frequency of the EEG signal (7–30 Hz), and the horizontal axis depicts time (seconds) relative to instruction onset. The inset color scale depicts the log ratio power values of the z-axis with significant clusters outlined in black (Patient 1, *p* = 0.018; Patient 3, *p* = 0.004). CRS-R, Coma Recovery Scale-Revised; VS, Vegetative State; MCS, Minimally Conscious State; OPJ, occipito-parietal junction; SMA, supplementary motor area; PMC, premotor cortex; PHG, parahippocampal gyrus; ERD, event-related desynchronization.

A summary of the results of the behavioral, EEG, and fMRI assessments is detailed in Table 2 and Figure 1. Patient 1 was diagnosed as in the VS from 21 CRS-R evaluations conducted in the 24-months prior to the fMRI/EEG assessments (highest score: 7; range: 4–7). In the fMRI study, this patient produced reliable and appropriate activation in both the spatial navigation and motor imagery tasks (occipito-parietal junction and supplementary motor area respectively, FWE *p* < 0.05). fMRI data for this patient have been previously reported (Fernández-Espejo and Owen, 2013). In the EEG task, this patient produced a contralateral ERD in the mu frequency band (9–13 Hz) for the conventional imagery (*p* = 0.018). Patient 2′s highest CRS-R score was 8 (range: 4–8, period: five assessments in 4 months), leading to a diagnosis of VS. Patient 2 did not produce reliable activation in the fMRI tasks or reliable spectral changes in the EEG tasks. Patient 3 scored in the MCS *Plus* range (highest score: 13, range: 11–13, period: four assessments in 4 months). This patient did not produce significant activation during the fMRI tasks. However, he did produce appropriate, reliable spectral changes during the conventional EEG motor imagery task (contralateral ERD, 7–13 Hz, *p* = 0.004). Patient 4 also scored in the MCS *Minus* (highest score: 10, range: 8–10, period: three assessments in 9 months). Although this patient showed no activation at the conservative FWE-corrected statistical threshold, she produced reliable, appropriate activation during the fMRI spatial navigation task in the bilateral occipito-parietal junction at an uncorrected *p* < 0.001. However, she did not produce reliable, appropriate activation for the fMRI motor imagery task, or the EEG motor imagery tasks. Patient 5 scored in the VS range (highest score: 6, range: 3–6, period: four assessments in 5 months) and did not produce reliable responses for any of the fMRI or EEG assessments. Finally, Patient 6 also scored in the VS range (highest score: 5, range: 3–5, period: four assessments in 3 months). As with Patient 4, reliable, appropriate activation was detected during the spatial navigation fMRI task (right parahipocampal gyrus and right premotor cortex, FWE *p* < 0.05). However, this patient did not produce reliable activation for the fMRI motor imagery task, or the EEG motor imagery tasks.

**Table 2:** **Behavioral, EEG, and fMRI assessment data**.

**Patient No**.	**Behavior**		**fMRI**		**EEG**	
	**Diagnosis (CRS-R Score^a^)**	**Command following**	**Spatial navigation**	**Motor imagery**	**Conventional Motor imagery**	**Familiar motor imagery**
1	Vegetative state (7)	No	Yes	Yes	Yes	No
2	Vegetative state (8)	No	No	No	No	No
3	Minimally conscious state *Plus*^b^ (13)	Yes	No	No	Yes	No
4	Minimally conscious State* Minus* (10)	No	Yes	No	No	No
5	Vegetative state (6)	No	No	No	No	No
6	Vegetative state (5)	No	Yes	No	No	No

*Abbreviations: CRS-R, Coma Recovery Scaled-Revised; fMRI, functional Magnetic Resonance Imaging; EEG, electroencephalography*.

a *Highest CRS-R score recorded by the research team prior to the time of assessment. For Patients 2-6, this period was 3–9 months (see text for details). For Patient 1, this period was 24 months (21 evaluations)*.

b *Patient 3 generated reproducible movements to spoken commands on the auditory sub-scale of the CRS-R on every evaluation*.

In summary, six patients were evaluated using a standard clinical behavioral assessment (the CRS-R; Kalmar and Giacino, 2005), two fMRI imagery tasks, and two EEG motor imagery tasks. Patient 1 (VS) was unable to follow commands behaviorally, but exhibited evidence of command following in both the fMRI and EEG tasks. Two patients (Patient 4 [MCS *Minus*] and Patient 6 [VS]) also showed no signs of behavioral command-following, but produced evidence of covert command following in the spatial navigation fMRI task. Patient 3 (MCS *Plus*) demonstrated evidence of command following both behaviorally and in the EEG conventional motor imagery task. Finally, Patients 2 (VS) and 5 (VS) did not demonstrate evidence of command following in either the behavioral, fMRI, or EEG assessments.

## Discussion

Functional neuroimaging methods for the detection of covert command-following have the potential to improve diagnostic and prognostic accuracy in DOC (for a review, see Owen, 2013). Due to the heterogeneity of etiology and pathology in this patient group, however, multiple imaging techniques and functional tasks are necessary to accurately identify a covert ability to follow commands. Here we provide the first report of the relative convergence and divergence of fMRI and EEG assessments of covert command-following in a small sample of patients with DOC.

Appropriate and statistically reliable signs of covert command-following were observed in three patients who were unable to follow commands with their behavior. Two of these patients (Patients 1 and 6) were repeatedly diagnosed as in the VS, adding to the growing body of evidence that the level of awareness possessed by severely brain-injured patients is not necessarily reflected in their external behaviors (for a review, see Owen, 2013).

Positive evidence for motor imagery command following was observed in the fMRI and EEG of one patient diagnosed as in the VS (Patient 1)—i.e., “Imagine playing tennis” in the fMRI, and “Imagine squeezing your right-hand” in the EEG. The covert awareness of this patient has been previously reported in other fMRI and EEG tasks (Cruse et al., 2012; Fernández-Espejo and Owen, 2013; Naci and Owen, 2013). The convergence of multiple assessment techniques in this way is one means by which confidence in the outcomes of individual assessments may be increased for each patient (Cruse et al., 2014). Indeed, across all published studies, this patient demonstrated their covert awareness with three separate fMRI tasks and two EEG tasks, thus providing perhaps unequivocal evidence that he was aware despite the outcomes of repeated clinical evaluations across his 12-years post-injury, and the 21 CRS-R assessments conducted across the two years in which he was enrolled in the research study.

Two patients (Patient 4 [MCS *Minus*] and Patient 6 [VS]) only demonstrated evidence of command following in the spatial navigation fMRI imagery task. The absence of significant results in the EEG tasks for these patients is consistent with the absence of significant activation during the fMRI “tennis” task in the same patients, as both require the engagement of motor imagery to command. From their radiological findings, Patient 6 (VS) presented with specific damage to motor areas as evident from scattered areas of low FLAIR signal and high T1 signal in the posterior precentral gyrus. Patient 1, on the other hand—also VS but, unlike Patient 6, capable of successfully performing the motor imagery tasks—showed no apparent damage to motor areas bilaterally. Together, these results suggest that the absence of reliable motor imagery responses in Patient 6, and potentially many other patients, may be a result of a specific impairment in motor injury, or at least in the detectability of its EEG/fMRI markers. Patient 4 (MCS *Minus*) also returned significant spatial navigation results only, but did not present with any specific motor area damage. However, this patient was tested 20-years post-injury and so it is possible that functional reorganization may have occurred in this time, although why this would be the case for motor imagery and not spatial navigation imagery is unclear. Nevertheless, the results of Patients 4 and 6 together emphasize the importance of presenting a battery of assessments of covert awareness in order to form the most accurate picture of a given patient's abilities. Indeed, if motor imagery were the only option provided to Patients 4 and 6, their covert level of awareness may never have been elucidated.

Patient 3 (MCS *Plus*) was both able to follow simple behavioral commands and return positive evidence of covert command-following in the EEG conventional motor imagery task. However, neither fMRI imagery task yielded positive results. While the presence of awareness was never under question for this patient due to their behavioral diagnosis of MCS *Plus*, the divergent fMRI and EEG results again highlight the importance of employing multiple modalities and tasks in the assessment of patients with DOC. Indeed, the fMRI and EEG assessments were performed on different days and at different times of the day, thereby increasing the patient's opportunities to demonstrate their command-following capacities. Moreover, varying levels of arousal and awareness are defining traits of patients in a MCS (Giacino et al., 2002) and may have contributed to the divergence between behavior and fMRI in this case.

Patients 2 and 5 (both VS) did not demonstrate evidence of command following in either the fMRI or EEG assessments, mirroring the outcomes of their behavioral evaluations. As has been discussed at length in previous work, null neuroimaging findings in this patient group cannot be interpreted as evidence that the patients lack awareness (Owen et al., 2006; Boly et al., 2007). Indeed, false negatives may occur in patients from fatigue, lack of understanding, or insufficient cognitive resources. Moreover, false negatives occur in neuroimaging studies of individuals without brain injury (Cruse et al., 2011; Naci et al., 2013; Fernández-Espejo et al., 2014). Both healthy volunteers and patients with brain injury may elect not to engage in an imagery task, making it impossible to distinguish negative findings that arise from a lack of ability from those due to an intention not to perform the task.

When a reliable and purposeful action has been identified in a standard behavioral assessment, a communicative interface may be formed (Gill-Thwaites and Munday, 1999; Kalmar and Giacino, 2005). For example, the CRS-R (Kalmar and Giacino, 2005) recommends tailoring the behavioral command-following tasks to the physical capacities of the patient, and even attempting multiple types of command. Any reliable responses that are observed to these commands may then be employed as a communicative output—e.g., “raise your finger for *yes*.” Similarly, covert actions detected with functional neuroimaging may be exploited for communication (Monti et al., 2010; Fernández-Espejo and Owen, 2013; Naci and Owen, 2013). In keeping with the behavioral approach, therefore, the application of multiple covert command-following tasks will not only provide the best opportunity for a patient to demonstrate their awareness, but may also provide them with a communicative outlet for the first time since their injury.

The current study also included an exploratory secondary EEG motor imagery task. All patients were asked to perform two types of motor imagery during the EEG task: (1) imagining squeezing their right-hands in line with conventional motor imagery EEG tasks (Pfurtscheller and Neuper, 1997; Cruse et al., 2011, 2012), and (2) imagining another familiar action that was selected by their caregivers. When a familiar action could not be identified, patients were asked to imagine dialing on a telephone, based on evidence for more robust brain responses to motor imagery involving finger sequencing actions (Roosink and Zijdewind, 2010; Gibson et al., 2013). The secondary task was motivated by the results of previous work with non-brain-injured volunteers (Curran and Stokes, 2003; Curran et al., 2004; Gibson et al., 2013). For example, experienced athletes and musicians produce more focused and reliable patterns of brain activation when they imagine actions involving the sport or instrument with which they have experience (Lotze et al., 2003; Wei and Luo, 2010). Nevertheless, there were no positive results for any patient in the familiar imagery task. It is possible that the apparently lower sensitivity of familiar imagery observed here is due to more variable brain responses that were not readily detectable with EEG. For example, imagined familiar actions may have involved memories or emotions to a greater extent than the more circumscribed hand squeezes. Indeed, focusing on the kinesthetic aspects of motor imagery is crucial in order to produce sensorimotor changes that can be observed in the EEG (Neuper et al., 2005). Although further study with a larger cohort is required before strong conclusions can be made, the current results suggest that instructing patients to imagine a familiar action with EEG is less sensitive than instructing them to imagine squeezing their hands into a fist. It is reassuring, however, that during the right-hand squeeze motor imagery task, both positive patients (Patients 1 and 3) produced contralateral ERDs in the mu band—a pattern that is consistent with previous studies of healthy individuals (Pfurtscheller and Neuper, 1997; Pfurtscheller and Lopes da Silva, 1999).

Detecting covert signs of awareness can improve diagnostic and prognostic accuracy in patients with DOC (see Owen, 2013, for a review). The current findings demonstrate that a range of tasks and neuroimaging modalities are required in order to accurately define the level of awareness possessed by patients with DOC. Indeed, two patients failed to return evidence of command-following in motor imagery tasks, but produced appropriate activation in a spatial navigation task. In these cases, the patients' specific patterns of brain damage may have disproportionately impaired some cognitive abilities or made their neural markers more difficult to observe. An effective battery of assessments for patients with DOC therefore should include a variety of tasks that probe a range of cognitive abilities supported by spatially-distinct brain regions and indexed by multiple neural signatures—e.g., EEG oscillations, event-related potentials, fMRI-detected hemodynamic responses, etc. Indeed, five patients were excluded from the current study because they did not qualify for evaluations with one of the two neuroimaging techniques. While no neuroimaging-based task will be 100% sensitive alone (e.g., Cruse et al., 2011; Gibson et al., 2013; Fernández-Espejo et al., 2014), the implementation of a battery of assessments alongside standardized behavioral evaluations will go a long way toward addressing the currently low rate of diagnostic accuracy for patients with DOC (Childs et al., 1993; Schnakers et al., 2009), and may open more avenues for two-way communication.

## Author contributions

Raechelle M. Gibson and Damian Cruse designed and conceived of the EEG imagery studies, collected and performed the statistical analyses of the EEG data, and drafted the manuscript with Davinia Fernández-Espejo and Laura E. Gonzalez-Lara. Davinia Fernández-Espejo collected and performed the statistical analyses of the fMRI data; Laura E. Gonzalez-Lara performed the behavioral assessments and assisted with the fMRI imagery data collection; and Benjamin Y. Kwan and Donald H. Lee evaluated the structural MRI scans of the patients. Adrian M. Owen conceived of the fMRI study and provided critical feedback on the manuscript with Benjamin Y. Kwan and Donald H. Lee. All authors read and approved the final manuscript.

### Conflict of interest statement

The authors declare that the research was conducted in the absence of any commercial or financial relationships that could be construed as a potential conflict of interest.

## Supplementary material

Supplementary Figure 1 **Single subject patterns of activation in a sample of 14 healthy young adults**. The participants completed the same mental imagery task of imagining spatial navigation as in the current work and were scanned using the same 3 Tesla Siemens scanner (Magnetom Trio Tim, Siemens, Germany) as the patients in the current work (left panel). Reproduced with permission from D. Fernández-Espejo from the original open-source publication (Fernández-Espejo et al., 2014).

Supplementary Figure 2 **Single subject patterns of activation in a sample of 14 healthy young adults**. The participants completed the same mental imagery task of imagining playing tennis as in the current work and were scanned using the same 3 Tesla Siemens scanner (Magnetom Trio Tim, Siemens, Germany) as the patients in the current work (left panel). Reproduced with permission from D. Fernández-Espejo from the original open-source publication (Fernández-Espejo et al., 2014).

Supplementary Figure 3 **Single subject patterns of event-related synchronizations and event-related desynchronizations over left and right motor cortex in a sample of six healthy young adults**. The participants completed the same conventional EEG motor imagery task as in the current work (right hand motor imagery) and an additional task of left hand motor imagery as indicated. Reproduced with permission from D. Cruse from the original open-source publication (Cruse et al., 2012).
