# Supplementary material for: Multiple tasks and neuroimaging modalities increase the likelihood of detecting covert awareness in patients with disorders of consciousness
Source: Front Hum Neurosci. 2014 Nov 26;8:950. doi: 10.3389/fnhum.2014.00950 (PMC4244609; doi:10.3389/fnhum.2014.00950)
Supplement: Supplementary file 1 [file Table1.DOCX]

**Supplementary Table 1. Familiar Motor Imagery Tasks.**

| **Patient No.** | **Familiar Imagery Task** |
| --- | --- |
|  |  |
| 1 | Make a tennis serve |
| 2 | Dial 9-1-1 |
| 3 | Lift a weight |
| 4 | Play a scale on the piano |
| 5 | Kick a soccer ball |
| 6 | Dial 9-1-1 |
