# Supplementary figures and images for: Multiple tasks and neuroimaging modalities increase the likelihood of detecting covert awareness in patients with disorders of consciousness
Source: Front Hum Neurosci. 2014 Nov 26;8:950. doi: 10.3389/fnhum.2014.00950 (PMC4244609; doi:10.3389/fnhum.2014.00950)

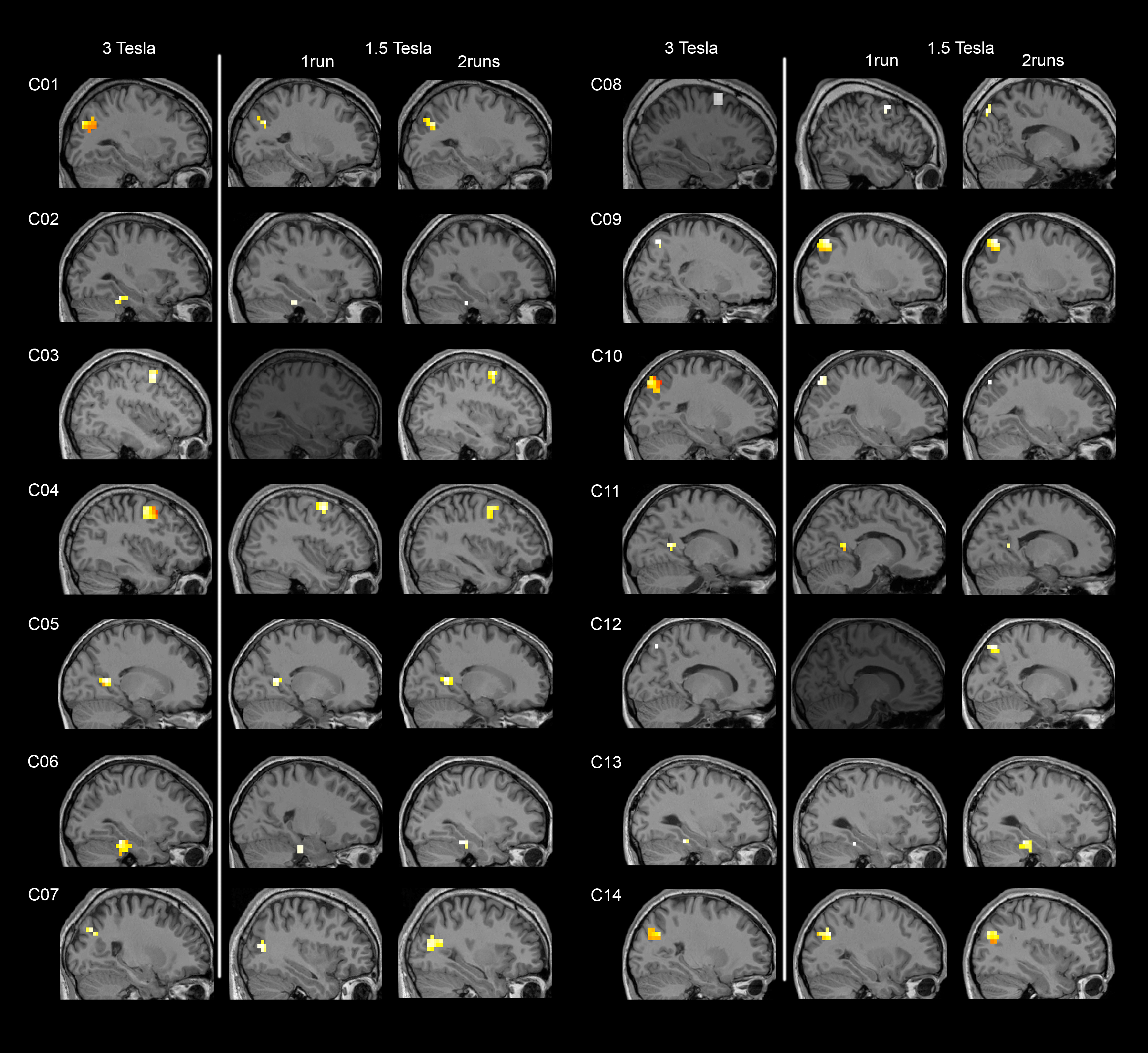

Supplement: Supplementary Figure 1 — Single subject patterns of activation in a sample of 14 healthy young adults. The participants completed the same mental imagery task of imagining spatial navigation as in the current work and were scanned using the same 3 Tesla Siemens scanner (Magnetom Trio Tim, Siemens, Germany) as the patients in the current work (left panel). Reproduced with permission from D. Fernández-Espejo from the original open-source publication (Fernández-Espejo et al., 2014). [file Image1.TIF]

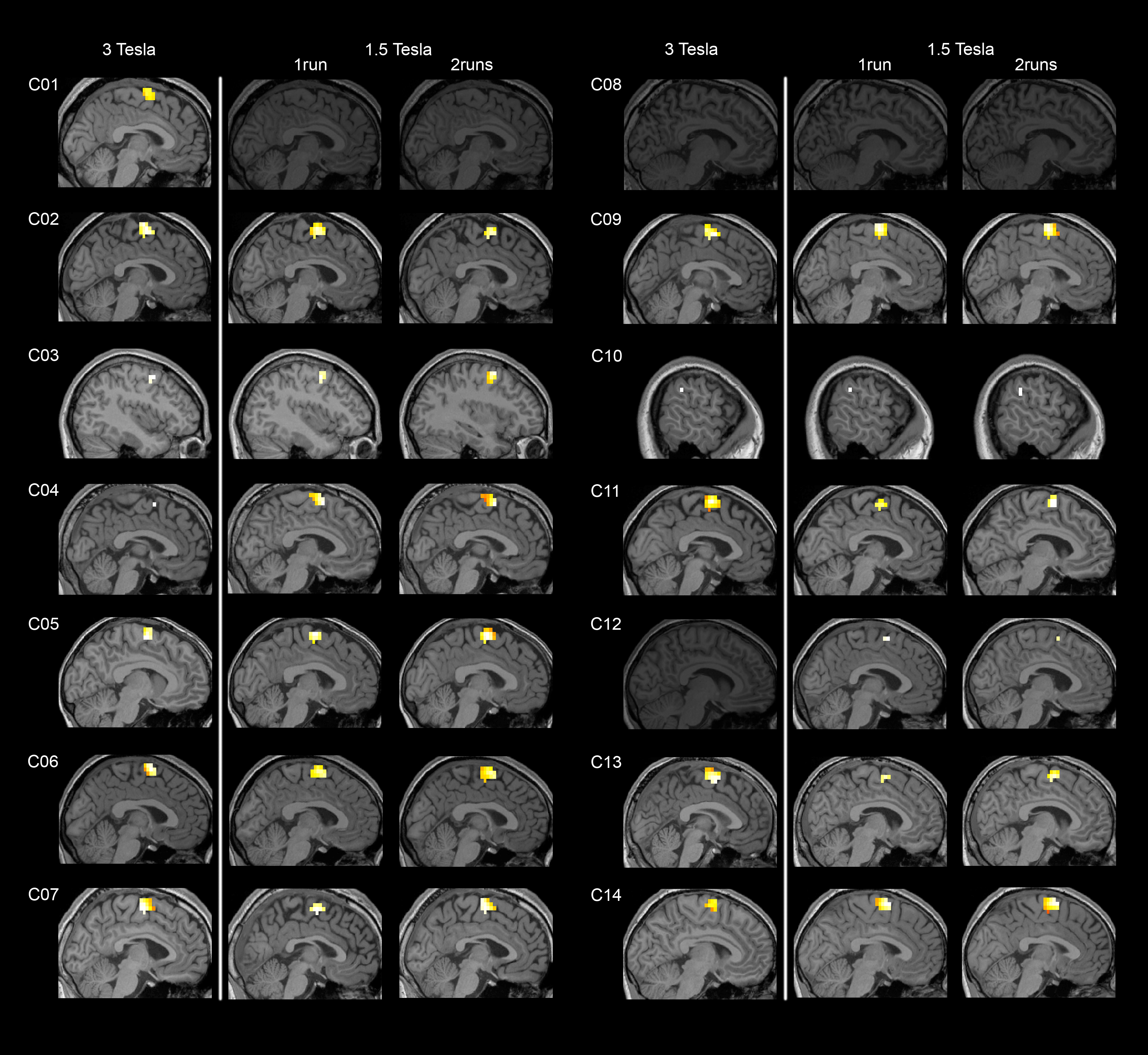

Supplement: Supplementary Figure 2 — Single subject patterns of activation in a sample of 14 healthy young adults. The participants completed the same mental imagery task of imagining playing tennis as in the current work and were scanned using the same 3 Tesla Siemens scanner (Magnetom Trio Tim, Siemens, Germany) as the patients in the current work (left panel). Reproduced with permission from D. Fernández-Espejo from the original open-source publication (Fernández-Espejo et al., 2014). [file Image2.TIF]

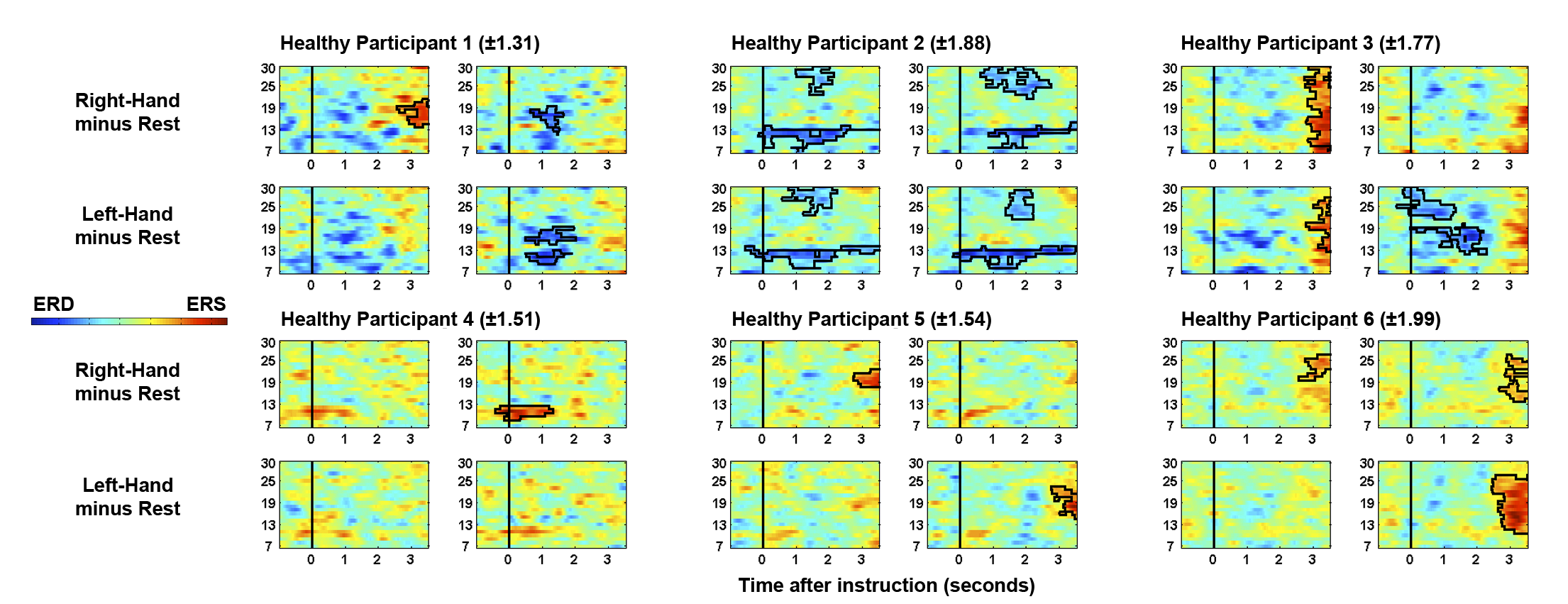

Supplement: Supplementary Figure 3 — Single subject patterns of event-related synchronizations and event-related desynchronizations over left and right motor cortex in a sample of six healthy young adults. The participants completed the same conventional EEG motor imagery task as in the current work (right hand motor imagery) and an additional task of left hand motor imagery as indicated. Reproduced with permission from D. Cruse from the original open-source publication (Cruse et al., 2012). [file Image3.TIF]
